# Vibrotactile Perception in Finger Pulps and in the Sole of the Foot in Healthy Subjects among Children or Adolescents

**DOI:** 10.1371/journal.pone.0119753

**Published:** 2015-04-02

**Authors:** Lars B. Dahlin, Nuray Güner, Helena Elding Larsson, Toni Speidel

**Affiliations:** 1 Department of Translational Medicine—Hand Surgery, Lund University, Skåne University Hospital, Malmö, Sweden; 2 Region Skåne Competence Center, Skåne University Hospital, Lund, Sweden; 3 Department of Clinical Sciences Malmö—Paediatric Endocrinology, Skåne University Hospital, Malmö, Sweden; 4 PID AB, Malmö, Sweden; University of Reading, UNITED KINGDOM

## Abstract

**Aims:**

To evaluate vibrotactile perception at different frequencies in fingers and in foot in healthy girls and boys.

**Methods:**

Vibration perception thresholds (VPTs) were measured in 283 healthy (8–20 years), consecutively included, girls (n=146) and boys (n=137); i.e., 269 children after excluding those with diseases or disorders possibly affecting the nervous system. Thresholds were measured in finger pulps of index and little fingers (seven frequencies; 8–500 Hz) and at first and fifth metatarsal head and at heel in the sole of the foot (six frequencies; 8–250 Hz;) using Multi Frequency Tactilometry.

**Results:**

VPTs, divided in six groups by age and gender (i.e., 8–10 years, 11–15 years and 16–20 years), at all three sites in the sole increased with higher frequencies, but without gender differences. Thresholds at 64 and 125 Hz were generally higher at heel compared to metatarsal heads. VPTs in finger pulps of index and little fingers, with no finger differences, had a different pattern with increasing thresholds with frequency, but with lower thresholds at 64 and 125 Hz. Thresholds at lower frequencies were higher in finger pulps, while at higher frequencies VPTs were lower in finger pulps than in the sole of the foot; thus, vibration perception in the sole was better than perception in finger pulps at lower frequencies and opposite at higher frequencies. VPTs were higher among adolescents than in younger children in the foot, while thresholds were lower in the finger pulps among adolescents, particularly in index finger. Thresholds in finger pulps of index and little fingers, particularly at higher frequencies, correlated with each other, which the three sites in the sole also did.

**Conclusions:**

VPTs in fingers and in feet are different as related to frequency in healthy girls and boys. Multi Frequency Tactilometry is a future valuable method to detect neuropathy in children and adolescents.

## Introduction

Vibrotactile perception at tactile surfaces in hands and feet, particularly in finger pulps and in the sole, are important for function [1,2], and may be impaired early in various neuropathies [3]. Vibrotactile perception depends on function of Paccini corpuscles, responding to frequencies > 80 Hz (probably in particular at 250 Hz [4]), and on Meissner´s corpuscles, which are most sensitive at 30 Hz [5,6]. Thus, to detect vibration perception thresholds (VPTs) at different frequencies, reflecting any dysfunction in such receptors and their connected axons, may be a useful tool for detection of various pathological conditions. In clinical practice, vibrotactile perception is commonly investigated at the pre-tibia, at the medial malleolus and at the big toe at a specific frequency to detect neuropathy [7,8], but tactile surfaces may be more important sites to examine as related to function [8,9,10]. Disturbed vibrotactile perception in finger pulps in vibration-induced neuropathy and in carpal tunnel syndrome has also been reported [3,11]. In the long-term, the development of neuropathy in sub-cohorts of population-based cohorts may be an important issue [8]. However, few studies have focused on the development of neuropathy in children with diabetes and other conditions and, if so, non-tactile surfaces have been examined [12,13,14]. Interestingly, recent data imply that vibrotactile sensory modality may also be one key to motor coordination difficulties of children with a developmental coordination disorder, where young children react more slowly [15]. To investigate occurrence of any neuropathy or disorder in children, VPTs have to be determined in a healthy population of children below the age of 20 years, since available data indicate that threshold changes are affected by aging and gender [16,17,18]; thus, such natural changes must be considered when making comparisons between groups or individuals. In addition, vibrotactile perception at tactile surfaces has not been examined in previous studies to obtain normative data in children and adolescents [19,20]. Our aim was to evaluate VPTs at tactile surfaces in finger pulps of the hand and in the sole of the foot unilaterally in healthy children and adolescents.

## Subjects and Methods

### Subjects

In total 283, consecutively included by invitation, girls (n = 146) and boys (n = 137) at the age of 8–20 years from three different schools, where we previously had established contacts, were invited to participate in the study after written information was provided to the parents and to the subjects. All children, irrespective of any tentative concomitant disease, from the different classes were invited to participate without any exclusion criteria and only a few declined. For ethical reasons we have no information of “non-participants”. Among the consecutively included 283 subjects, 14 subjects were excluded from further analyses, due to diabetes, cognitive disorders, obvious incapability to understand the instructions (n = 6 girls and n = 6 boys), or incomplete recordings (n = 2; one of each gender).

### Set up for examination of fingers

**Fig 1:**
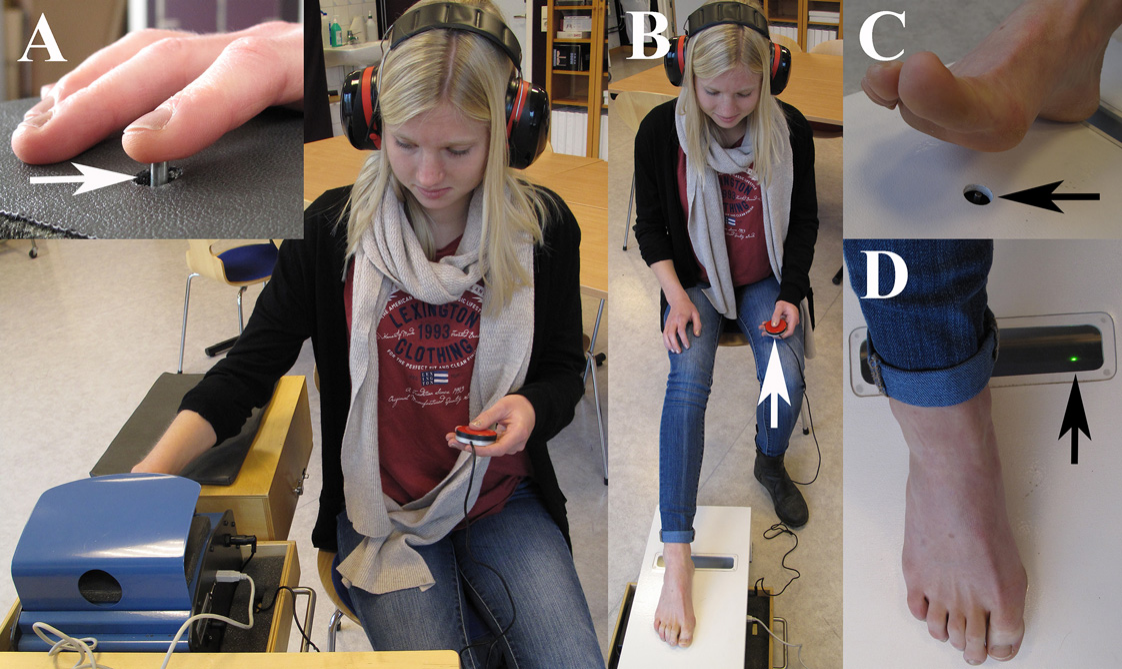
Photo showing the experimental set-up. The subject is sitting with the arm/hand and leg/foot, respectively in a relaxed position (A). The investigated skin area (index or little finger, head or the first or fifth metatarsal bone or heel) is placed on a vibrating probe (insert). The subject regulates the intensity of the vibration by pressure a button on a remote control (arrow) in his/her contralateral hand (B). The result is a tactilogram (see Fig. 2). The probe for the foot is shown in (C) and the green light (D) indicates that the pressure is appropriate on the investigated area. The person on the photo is *not* one of the subjects in the study.

Vibration perception thresholds (VPTs) were measured in the right hand at the pulp of the index and little fingers with a standard VibroSense Meter device. The set up for measuring VPTs on finger pulps was done according to ISO 13091–1, Method A; i.e. without a surround and a contact force of 0.15 ± 0.09 Newton (corresponding to a static skin indent of about 1.5 mm) between the probe and the finger pulp. The probe diameter was 4 mm. The skin contact force was continuously monitored by the operator to make sure that the contact force was within required limits according to ISO 13091–1. During the examination the subject was seated comfortable in a chair (Fig. 1). The examined finger was placed on the vibrating probe, which was covered with a shield to prevent the subject to have a visual contact with the probe. Each examination round started with a test recording at 16 Hz in only one finger; thus, the subject could be acquainted with the procedure. The examination sequence was the index finger followed by the little finger. We only tested VPTs unilaterally since previous studies have not indicated any difference between the right and left side.

**Fig 2:**
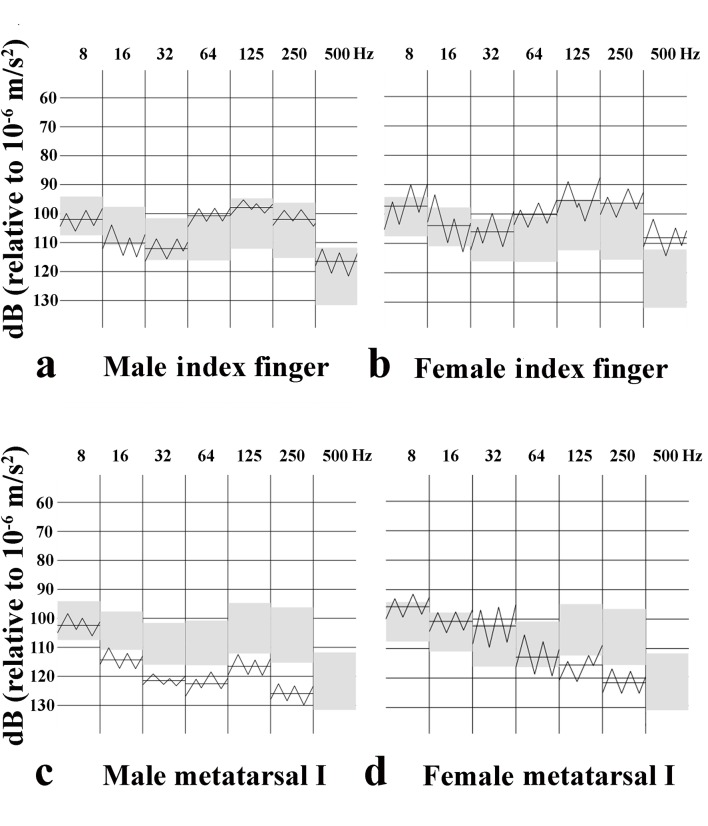
Tactilogram with vibrotactile perception thresholds. Vibrotactile perception thresholds (VPTs) at different frequencies from a male (A, C) and female (B, D) in the index finger (A, B) and at the first metatarsal (metatarsal I) head in the sole of the foot (C, D). The grey area corresponds to normative age mapped VPTs values (mean ± 1 SD) for healthy adults.

### Set up for examination of foot

VPTs were also measured at the sole of the foot at three different locations; i.e. at the first and fifth metatarsal head as well as on the heel with a modified VibroSense Meter adopted for measurement on the feet (Fig. 1). The foot VibroSense Meter comprised of a modified hand device with an extended vibrating probe and a special footplate with a hole, which was placed over the modified device. The modified hand device was mounted on a cradle, which could be adjusted in height in order to achieve a proper contact force between the probe and the skin on the examined body part. Hence, the investigated location of the sole of the foot (i.e. head of the first and fifth metatarsals or heel) was placed on the footplate above the hole through which the extended probe was protruding. The skin contact force was controlled by the operator, who adjusted the height of the cradle before starting the examination of VPTs. The measuring procedure for the foot was identical with the examination if the fingers; i.e. according to ISO 13091–1. During the examination the subject was seated comfortable in a chair putting the right foot on the footplate with an angle between the thigh and the lower leg of about 90 degree. The examined site was placed on the vibrating probe and the subject did not have visual contact with the probe since the foot covered it. Each examination round started with a test recording at 16 Hz in only one site; thus, the subject could be acquainted with the procedure. The examination sequence was first metatarsal head, fifth metatarsal head and heel (time taken to examine: approximately 3 min/site as in the fingers).

### Examination procedure

The operator explained the examination procedure to the subject. The instruction was very simple; i.e. press and keep the response button down when you can perceive a vibration and release the button when no vibration is perceived. The VPTs were measured with Multi Frequency Tactilometry (Fig. 1) in accordance with previously described technique [1,9,10,11]. The VibroSense Meter device was controlled by a connected standard PC, running the VSM software (version 1.46.6; proprietary software), which is a part of the VibroSense Meter system. The subject was provided with hearing protection to avoid any acoustic impulses and to facilitate concentration. The applied vibrations were controlled according to a von Békésy up/down psychophysical algorithm [1,9] and the amplitude (acceleration) ramp rate was 3 dB/s, relative to 10^-6^ m/s^2^. Prior to the examination the temperature was measured with the internal temperature probe on the VibroSense Meter, which is a standard procedure for the VSM device at each investigated extremity to make sure that is was in the appropriate interval, i.e. 27°-35°, which is according to ISO 13091–1 [21,22,23]. The room temperature was between 20°-22°C (requirement according to ISO 13091–1).

The probe, vibrating at specific frequencies, excited the investigated areas in the finger pulp or in the sole of the foot. The thresholds were measured in one hand in each subject in the finger pulps of index and little fingers at seven frequencies (8, 16, 32.5, 64, 125, 250 and 500 Hz) as well as at six frequencies in one foot at three sites in the sole of the foot (8, 16, 32.5, 64, 125 and 250 Hz; first and fifth metatarsal head and at heel). Thresholds were not measured at 500 Hz in the foot since the required power exceeded the specification limits for the VibroSense Meter equipment [10].

The applied acceleration [expressed in decibels (dB)] on the probe, exciting the finger pulp or sole of the foot, started at 80 dB and then increased with a speed of 3dB/s until the subject perceived a vibration whereby the subject pressed a hand switch; i.e. the response button. The intensity then decreases with a speed of 3dB/s until the subject no longer can feel the vibrations whereby the subject releases the switch. The procedure is repeated four times for each frequency.

A VPT is calculated as the mean value of the three last upper and lower perception levels, i.e. the first upper and lower perception level is discarded in compliance with ISO 13091–1.

The tactilometry examination is fully automated without any manual setting of frequency or vibration levels, indicating that any person (i.e. physician, nurse or BMA) can run the system. Thus, the equipment controls all frequencies and vibration levels where thresholds are examined from low to high frequency. The examination takes approximately three minutes per site and the same operator did all measurements. Data from each patient were stored in a database by the Vibrosense Meter PC software. [1,9,24,25]. Height and weight were also measured in all subjects.

### Ethics statement

The local ethics committee at Lund University approved the study (386/2007). Written informed consent was obtained from the parents of the children and adolescents <18 years and from each subject >18 years. The study was conducted in accordance with the declaration of Helsinki (http://www.wma.net/en/20activities/10ethics/10helsinki/).

### Statistical analyses

Values of VPTs from the five different sites are expressed as means (95% confidence intervals) and with comparisons as mean differences (95% confidence intervals). The vibrations thresholds were divided by age from 269 children, where thresholds were obtained [i.e. 8–10 years (41 girls, 36 boys), 11–15 years (55 girls, 54 boys) and 16–20 years (43 girls, 40 boys)]. Statistical differences were tested by the Student´s *t*-test (paired samples *t*-tests). We only tested those differences where the confidence intervals indicated a visual difference between groups. Only one subgroup was tested, which consisted of 20 comparisons. Therefore, with Bonferroni method the alpha-level value was adjusted to 0.0025. Correlations between measurements in index and little finger, first and fifth metatarsal heads, first metatarsal head and heel and between fifth metatarsal head and heel at the different frequencies were determined by Pearson correlation matrix (rho>0.3 required and p<0.05). The statistical analyses were done using IBM SPSS Statistics (Statistical Package for the Social Sciences, SPSS Inc., Chicago, Il, USA) version 20 for Mac.

## Results

### Demographics of subjects

The VPTs were divided by age from 269 children, where thresholds were obtained [i.e. 8–10 years (41 girls, 36 boys), 11–15 years (55 girls, 54 boys) and 16–20 years (43 girls, 40 boys)]. Height and weight did not differ between girls and boys among the lower two age classes, but values of height and weight were higher among boys at the highest age class (Table 1).

**Table 1:** Height and weight of the 269 healthy children included in the study.

Gender	Number	Age (years)	Height (cm)	Weight (kg)
**Girls**	41	8–10	137.7 (134.5–140.9)	33.8 (30.9–36.7)
	55	11–15	157.6 (155.3–159.9)	48.5 (45.9–51.1)
	43	16–20	167.8 (166.1–169.4)	58.8 (56.8–60.8)
**Boys**	36	8–10	137.1 (134.1–140.1)	32.9 (30.5–35.3)
	54^a^	11–15	158.0 (155.2–160.1)	48.4 (44.5–56.3)
	40	16–20	179.4 (176.5–182.3)	67.7 (64.0–71.4)

Values are means and 95% confidence intervals in parenthesis.

^a^ Data missing in two subjects.

### Vibration perception thresholds

#### Sole of the foot

VPTs at all three sites in the sole of the foot showed a clear trend, i.e. unnecessary with p-values, with increasing thresholds at higher frequencies, but with no differences between girls and boys (Tables 2, 3 and 4 and Fig. 2). The thresholds were generally higher at the heel at 64 and 125 Hz compared to thresholds at the metatarsal heads [p<0.0001, i.e. mean difference (95% confidence intervals): 64 Hz heel-first metatarsal, 5.74 (95% CI: 4.8–6.7); 64 Hz heel-fifth metatarsal, 5.54 (95% CI: 4.7–6.4); 125 Hz heel-first metatarsal, 8.11 (95% CI: 7.0–9.2); 125Hz heel-fifth metatarsal, 7.78 (95% CI: 6.8–8.7)] for all subjects (i.e. heel compared to first and fifth metatarsal, respectively without division in age or gender). In addition, the thresholds were higher among the adolescents than in the younger children, particularly at 64 Hz [i.e. among girls at heel: 64 Hz p<0.0001, mean differences -6.03 (95% CI: -9.0-(-3.0); 32 Hz p = 0.0002, -4.21 (95% CI: -6.8-(-1.6); no differences at first and fifth metatarsals; and among boys at heel: 64 Hz p = 0.001, -5.48 (95% CI: -8.5-(-2.5)) and 32 Hz p = 0.025, -3.57 (95% CI: -6.7-(-0.5)); at first metatarsal 64 Hz: p = 0.002, -5.69 (95% CI: -9.3-(-2.1)) and 32 Hz p = 0.12, -2.61 (95% CI: -5.9–0.7); at fifth metatarsal 64 Hz: p = 0.001, -6.43 (95% CI: -10.2-(-2.7)); 32 Hz p = 0.087, -2.60 (95% CI: -5.6–0.4); thus, no difference at 32 Hz, but difference at 64 Hz].

**Table 2:** Vibration perception thresholds at the heel in the sole of the foot at different frequencies in 269 healthy girls and boys.

Heel								
Gender	Number	Age (years)	8 Hz	16 Hz	32 Hz	64 Hz	125 Hz	250 Hz
**Girls**	41	8–10	96.9 (94–99)	103.3 (101–106)	108.8 (106–111)	112.9 (110–115)	120.4 (118–122)	130.6 (128–133)
	55	11–15	97.6 (96–100)	103.7 (102–105)	109.7 (108–112)	115.4 (113–117)	120.3 (118–123)	130.9 (128–133)
	43	16–20	97.3 (96–99)	104.1 (102–106)	113.0 (112–114)	119.0 (117–121)	122.5 (120–125)	133.0 (130–136)
**Boys**	36	8–10	94.6 (89–101)	100.0 (94–106)	105.7 (99–112)	111.1 (104–118)	116.1 (109–123)	126.4 (118–134)
	54	11–15	98.7 (98–100)	104.0 (102–106)	111.5 (110–113)	118.1 (116–120)	120.8 (119–123)	128.6 (126–131)
	40	16–20	98.6 (97–100)	104.6 (103–106)	112.0 (110–114)	119.7 (118–122)	123.2 (121–126)	133.8 (130–138)

Values, expressed in dB (relative to 10^-6^ m/s^2)^, are means and 95% confidence intervals in parenthesis.

**Table 3:** Vibration perception thresholds at the first metatarsal head in the sole of the foot at different frequencies in 269 healthy girls and boys.

First metatarsal head								
Gender	Number	Age (years)	8 Hz	16 Hz	32 Hz	64 Hz	125 Hz	250 Hz
**Girls**	41	8–10	96.0 (94–98)	101.0 (99–102)	106.3 (104–108)	110.5 (108–113)	113.3 (111–116)	126.0 (123–130)
	55	11–15	98.0 (96–100)	102.5 (101–104)	106.5 (105–108)	109.6 (107–112)	111.0 (109–113)	122.1 (120–125)
	43	16–20	97.5 (95–99)	103.7 (102–105)	108.6 (107–110)	111.8 (110–114)	115.0 (113–117)	127.4 (125–130)
**Boys**	36	8–10	97.8 (95–100)	101.5 (100–103)	106.6 (104–109)	107.3 (105–110)	111.2 (108–114)	122.7 (119–126)
	54	11–15	97.6 (96–99)	102.7 (101–104)	108.7 (107–111)	112.6 (110–115)	113.1 (110–116)	123.6 (121–126)
	40	16–20	98.0 (96–100)	102.9 (101–105)	109.3 (107–111)	113.0 (110–116)	114.5 (112–118)	126.5 (124–129)

Values, expressed in dB (relative to 10^-6^ m/s^2)^, are means and 95% confidence intervals in parenthesis.

**Table 4:** Vibration perception thresholds at the fifth metatarsal in the sole of the foot at different frequencies in 269 healthy girls and boys.

Fifth metatarsal head								
Gender	Number	Age (years)	8 Hz	16 Hz	32 Hz	64 Hz	125 Hz	250 Hz
**Girls**	41	8–10	97.0 (95–99)	102.6 (101–105)	106.4 (104–109)	109.8 (107–113)	113.7 (112–116)	127.3 (124–130)
	55	11–15	97.6 (96–99)	103.1 (102–105)	108.4 (107–110)	110.8 (109–113)	112.3 (110–115)	124.4 (122–127)
	43	16–20	97.7 (96–99)	103.2 (102–105)	108.9 (107–111)	111.3 (109–114)	114.5 (112–117)	128.6 (126–131)
**Boys**	36	8–10	96.7 (95–99)	101.5 (99–104)	106.1 (104–109)	107.7 (105–111)	110.3 (107–114)	122.6 (119–127)
	54	11–15	97.0 (96–98)	102.7 (102–104)	108.1 (107–109)	112.3 (110–115)	112.8 (110–115)	123.6 (121–126)
	40	16–20	96.9 (95–98)	102.7 (101–105)	108.7 (107–110)	114.1 (112–117)	116.2 (114–119)	130.1 (127–134)

Values, expressed in dB (relative to 10^-6^ m/s^2)^, are means and 95% confidence intervals in parenthesis.

#### Finger pulps of index and little fingers

**Fig 3:**
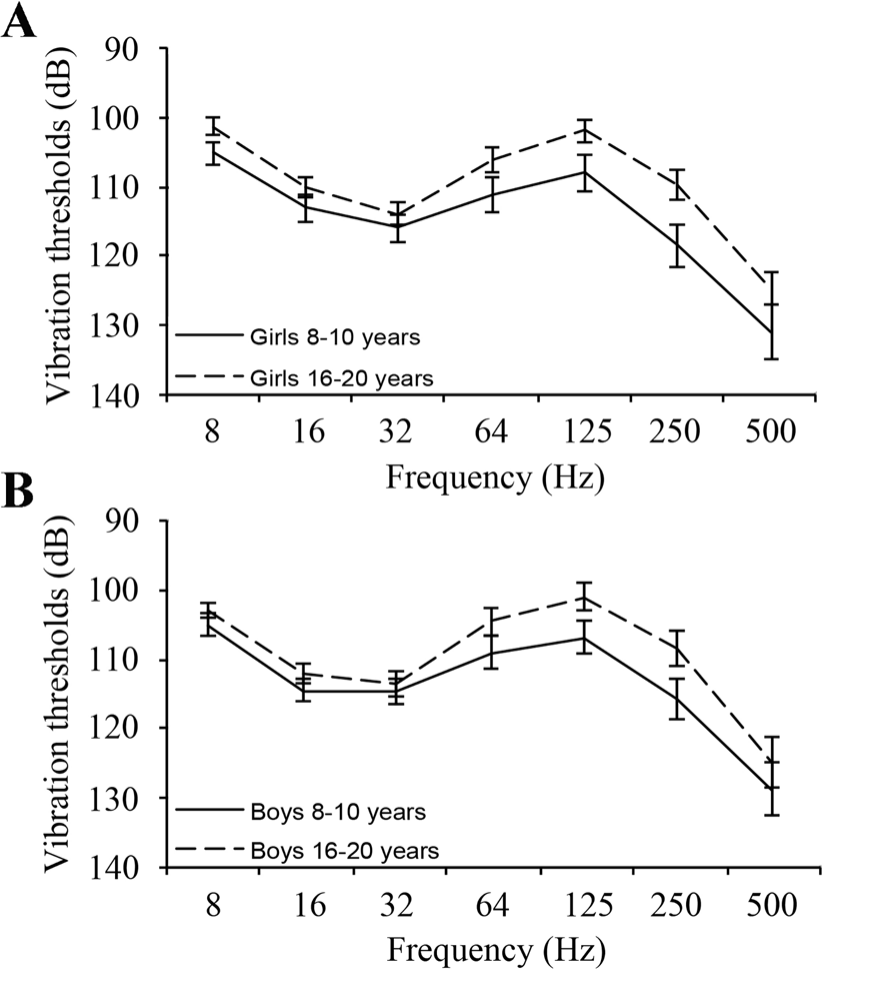
Spaghetti plots with vibration perception thresholds. Vibration perception thresholds at different frequencies from the index fingers in young (8–10 years) and older (16–20 years) children in girls (A) and boys (B). Values, expressed in dB (relative to 10^-6^ m/s^2^), are means and 95% confidence intervals.

VPTs in finger pulps of index and little fingers, which did not differ, had a different pattern with increasing thresholds with frequency, but lower thresholds at 64 and 125 Hz (Tables 5 and 6; Figs. 2 and 3; spaghetti plots). Thresholds were higher at low frequencies and lower at high frequencies in finger pulps than in the sole of the foot. Statistical differences were tested particularly at 32 Hz (i.e. Meissner´s corpuscles) and at 250 Hz (Paccini corpuscles). Paired comparisons showed differences between little finger and fifth metatarsal at 32 Hz [p<0.0001; 8.30 (95% CI: 7.5–9.1)] and at 250 Hz [p<0.0001; -14.9 (95% CI: -16.2-(-13.5)] as well as between index finger and first metatarsal at 32 Hz [p<0.0001; 6.89 (95% CI: 5.9–7.9)] and 250 Hz [p<0.0001; -12.29 (95% CI: -13.6-(-11.0)]. Furthermore, the thresholds were lower in the finger pulps among the adolescents than in the younger children mainly at the higher frequencies in the index finger [i.e. girls: 125 Hz: p<0.0001, 6.13 (95%CI: 3.0–9.2); 250 Hz: p<0.0001, 8.79 (95% CI: 5.1–12.4); 500 Hz: p = 0.006, 6.32 (95% CI: 1.9–10.8); boys: 125 Hz: p<0.0001, 5.79 (95% CI: 2.8–8.8), 250 Hz: p<0.0001, 7.36 (95% CI: 3.6–11.1); 500 Hz: p = 0.134, 3.91 (95% CI: -1.2–9.1); Fig. 2], but fewer differences were found in the little finger [e.g. girls 8 Hz: p<0.0001, 3.67 (95% CI: 1.7–5.6); 125 Hz: p = 0.001, 6.42 (95% CI: 2.6–10.2)], in which boys did not seem to express any differences as related to age.

**Table 5:** Vibration perception thresholds in the finger pulp of index finger at different frequencies in 269 healthy girls and boys.

Index finger									
Gender	Number	Age (years)	8 Hz	16 Hz	32 Hz	64 Hz	125 Hz	250 Hz	500 Hz
**Girls**	41	8–10	105.0 (103–107)	112.9 (111–115)	115.8 (114–118)	110.9 (108–114)	107.8 (105–110)	118.4 (115–121)	131.0 (127–135)
	55	11–15	111.4 (110–113)	111.4 (110–113)	115.6 (113–117)	106.8 (105–109)	103.2 (101–105)	111.8 (110–114)	126.0 (123–129)
	43	16–20	101.1 (100–102)	110.0 (109–111)	113.7 (112–115)	105.9 (104–108)	101.7 (100–103)	109.6 (107–112)	124.6 (122–127)
**Boys**	36	8–10	105.1 (103–107)	114.4 (113–116)	114.6 (113–117)	109.1 (107–111)	106.8 (104–109)	115.8 (113–119)	128.7 (125–132)
	54	11–15	102.8 (102–104)	111.4 (110–113)	114.3 (113–115)	107.3 (106–109)	105.6 (104–107)	110.9 (109–113)	124.2 (122–126)
	40	16–20	103.0 (102–104)	112.0 (111–113)	113.5 (112–115)	104.6 (103–107)	101.0 (99–103)	108.4 (106–111)	124.8 (121–128)

Values, expressed in dB (relative to 10^-6^ m/s^2)^, are means and 95% confidence intervals in parenthesis.

**Table 6:** Vibration perception thresholds in the finger pulp of little finger at different frequencies in 269 healthy girls and boys.

Little finger									
Gender	Number	Age (years)	8 Hz	16 Hz	32 Hz	64 Hz	125 Hz	250 Hz	500 Hz
**Girls**	41	8–10	105.4 (104–107)	111.9 (110–114)	117.1 (115–119)	110.9 (108–114)	108.7 (105–112)	116.7 (113–121)	127.4 (124–131)
	55	11–15	103.3 (102–105)	110.5 (109–112)	115.8 (114–117)	107.8 (106–110)	104.5 (102–107)	111.7 (109–115)	126.5 (124–129)
	43	16–20	101.7 (101–103)	109.2 (108–111)	114.9 (113–116)	106.6 (104–109)	102.3 (100–104)	108.6 (106–111)	124.7 (122–127)
**Boys**	36	8–10	105.9 (104–108)	112.3 (110–114)	116.7 (115–119)	109.3 (107–112)	105.9 (103–109)	112.3 (109–116)	125.9 (122–129)
	54	11–15	103.5 (102–105)	111.5 (110–113)	115.8 (114–117)	106.9 (106–108)	104.1 (103–106)	108.6 (107–111)	123.4 (121–125)
	40	16–20	103.9 (103–105)	110.2 (109–111)	116.7 (115–118)	107.0 (105–109)	102.4 (100–104)	109.3 (106–112)	123.7 (120–127)

Values, expressed in dB (relative to 10^-6^ m/s^2)^, are means and 95% confidence intervals in parenthesis.

### Correlations

The Pearson correlation matrix showed that there were correlations found between thresholds in finger pulps of index and little fingers, particularly at higher frequencies in both girls (Table 7) and boys (Table 8). In addition, correlations were also discovered between the three sites in the sole of the foot in the girls and the boys (Tables 7 and 8), except in boys between thresholds at the heel and the sites of metatarsal heads (Table 8).

**Table 7:** Pearson correlation (2-tailed) between vibration perception thresholds in index and little fingers as well as between three sites in the sole of the foot at different frequencies measured in 139 healthy girls.

Girls	8 Hz	16 Hz	32 Hz	64 Hz	125 Hz	250 Hz	500 Hz
**Index and little fingers**	0.51	0.56	0.62	0.76	0.76	0.75	0.73
**First and fifth metatarsal**	0.42	0.48	0.53	0.53	0.58	0.58	-
**First metatarsal and heel**	0.35	0.31	0.36	0.48	0.45	0.49	-
**Fifth metatarsal and heel**	0.59	0.53	0.51	0.64	0.58	0.46	-

Data are rho-values and only rho-values if rho>0.30 are presented. The rho-values all showed p<0.01.

**Table 8:** Pearson correlation (2-tailed) between vibration perception thresholds in index and little fingers as well as between three sites in the sole of the foot at different frequencies measured in 130 healthy boys.

Boys	8 Hz	16 Hz	32 Hz	64 Hz	125 Hz	250 Hz	500 Hz
**Index and little fingers**	0.46	0.54	0.46	0.50	0.67	0.65	0.72
**First and fifth metatarsal**	0.55	0.52	0.48	0.61	0.73	0.66	-
**First metatarsal and heel**	-	-	-	-	0.31	0.38	-
**Fifth metatarsal and heel**	-	-	-	-	0.39	0.51	-

Data are rho-values and only rho-values if rho>0.30 are presented. The rho-values all showed p<0.01.

## Discussion

Vibrotactile perception in finger pulps of healthy subjects with an age below 20 years showed differences at various frequencies and sites in the foot and the fingers as well as differences related to age. This indicates that normative data are needed for vibrotactile perception with focus on various sites and frequencies in girls and boys during childhood and adolescence. The sites were selected to mirror the function of the median (index finger), the ulnar (little finger), the median plantar branch of the tibial (first metatarsal head), the lateral plantar branch of the tibial (fifth metatarsal head) and the sural (heel) nerves. We did not intend to predict any specific factor; thus, without a specific expectation if and how the age could affect the vibration thresholds. The age of puberty in girls and boys is different and extends from 11 to 15 years [26]. We focused on a rough development of puberty and how that development influences the thresholds. We could not precisely define the exact stage of puberty for specific reasons (i.e. Tanner stage); thus, the 269 healthy children were divided into three classes; prepubertal (i.e. 8–10 years of age), pubertal (i.e. 11–15 years of age) and postpubertal (i.e. 16–20 years of age) stages [27]. Height and weight did not differ between the girls and the boys up to 15 years, but were higher in boys than in girls at 16–20 years. Interestingly, we observed that the VPTs in the sole of the foot were higher among adolescents than in younger children, while it was opposite in the hand, particularly in the index finger, where thresholds were lower among the adolescents. The former statement is in agreement with Olsen et al [14], who also found a positive correlation between age and VPTs at *one* frequency in the big toe, but only among healthy boys. However, no data have been presented concerning any correlation between age and thresholds in fingers or if tactile or non-tactile sites were examined [14]. Generally, few studies have focused on VPTs. If such an investigation has been done, only a single frequency has been tested and not at tactile surfaces [19,20,28,29]. The present site differences were demonstrated by higher VPTs at lower frequencies in finger pulps than in the sole, while the VPTs were lower in the finger pulps at higher frequencies than in the sole; thus, an indication that the vibration perception in the sole was better than the perception in finger pulps at lower frequencies and the opposite was seen at higher frequencies. In accordance with Hilz et al [19], thresholds at 125 Hz in the present study were higher in the foot than in fingers. However, the thresholds at low frequencies were lower in the sole of the foot. Thus, the differences depended on the site in the sole of the foot, which may reflect the need of such sense on function of foot and hand, which is understandable from a physiological point of view. Interestingly, Paccini corpuscles respond to frequencies above 80 Hz, probably in particular at 250 Hz [4], while Meissner´s corpuscles react to lower frequencies (particularly 30 Hz) [30,31].

An age- and gender-matched control group for vibrotactile perception has not previously been available for children, but has been published for adults, particularly for men [11]. Data indicate that VPTs in men and women are differently affected by diabetes with a higher risk for neuropathy in male patients with diabetes [9,14,32,33]. Interestingly, adult males have a lower density of intraepidermal nerve fibres (i.e. non-myelinated) than adult females in the hand [34], but no data is to our knowledge available for fibre density in children and adolescents. The development of the plastic brain may be another factor contributing to the present results of the variability between children and adolescents at some frequencies. Outcome of a peripheral nerve repair after injury is better if the injury has occurred below the age of 12 years; probably due to cerebral plasticity and not a better regenerative capability in young children [27]. Recent data imply that the vibrotactile sensory modality may be one key to the motor coordination difficulties of children with a developmental coordination disorder and that there may be a difference between younger and older children in responding to vibrotactile stimuli; the former reacting more slowly [15].

The strength of the present study was that we compared subjects at three different age groups, which all were healthy, but a limitation is that we did not include comparison with any previously used technique [19]. We only tested vibrotactile perception unilaterally since previous studies have not indicated any difference between the right and left side (i.e. dominant and non-dominant side). More importantly, we carefully checked the temperature of the foot and fingers before the measurement, since such a factor influences vibrotactile perception. Present normative data for children and adolescents may serve as a basis to detect neuropathy among children and adolescents with different disorders, particularly in diabetes in which neuropathic signs recently were reported [12,13,14]. It is relevant to detect a disturbed vibrotactile perception in children with type 1 diabetes since estimation from a smaller population indicates that almost half of the children with diabetes have subclinical large- and small-fibre neuropathies, even if tactile detection seems to be better than assessing vibrotactile perception [13]. Nevertheless, quantitative sensory testing is valuable and may be important when evaluating new intervention strategies and even body balance, particularly if tactile surfaces in hands and feet are investigated and multiple frequencies are utilized [19,29,35]. Interestingly, even children with toe-walking gait show different VPTs than controls, reflecting physiological changes in the localized receptors within the skin or at a neural perception level in these children [36]. In most of previous studies, the Biothesiometer has been used to detect vibrotactile perception, but again applied at non-tactile surfaces [12,28,37]. However, the gold standard is still electrophysiology, which is a more complicated method unsuitable for screening in clinical routine [12]. The age-dependence of VPTs has also been emphasized by others [20], although non-tactile surfaces were examined.

The vibration thresholds at each site were only measured once in each subject. It is less likely that training in an individual subject would influence results and conclusions due to the number of subjects. We are not entirely sure about the impact of training on individual thresholds with time. A combined score used in previous studies, i.e. sensibility index (SI; 22), may vary ± 0.03 if the examination is done within a short time and if done after several weeks ± 0.05. The technique should not be used everyday; probably not more frequently than once every month, which is actually relevant in clinical settings.

## Conclusions

Vibration perception thresholds in fingers and in the sole of the foot are different as related to frequency in healthy girls and boys, reflecting the physiological development during growth. Tactilometry is a future valuable method to detect neuropathy and is easy to handle.

## References

[1] Dahlin LB, Thrainsdottir S, Cederlund R, Thomsen NO, Eriksson KF, Rosèn I, et al (2008) Vibrotactile sense in median and ulnar nerve innervated fingers of men with Type 2 diabetes, normal or impaired glucose tolerance. Diabet Med 25: 543–549. 10.1111/j.1464-5491.2008.02433.x 18346156

[2] Cederlund RI, Thomsen N, Thrainsdottir S, Eriksson KF, Sundkvist G, Dahlin LB. (2009) Hand disorders, hand function, and activities of daily living in elderly men with type 2 diabetes. J Diabetes Complications 23: 32–39. 10.1016/j.jdiacomp.2007.09.002 18413154

[3] Thomsen NO, Cederlund R, Speidel T, Dahlin LB (2011) Vibrotactile sense in patients with diabetes and carpal tunnel syndrome. Diabet Med 28: 1401–1406. 10.1111/j.1464-5491.2011.03308.x 21480975

[4] Bell J, Bolanowski S, Holmes MH (1994) The structure and function of Pacinian corpuscles: a review. Prog Neurobiol 42: 79–128. 7480788 10.1016/0301-0082(94)90022-1

[5] Mountcastle VB, LaMotte RH, Carli G (1972) Detection thresholds for stimuli in humans and monkeys: comparison with threshold events in mechanoreceptive afferent nerve fibers innervating the monkey hand. J Neurophysiol 35: 122–136. 4621505 10.1152/jn.1972.35.1.122

[6] LaMotte RH, Mountcastle VB (1975) Capacities of humans and monkeys to discriminate vibratory stimuli of different frequency and amplitude: a correlation between neural events and psychological measurements. J Neurophysiol 38: 539–559. 1127456 10.1152/jn.1975.38.3.539

[7] Karvestedt L, Martensson E, Grill V, Elofsson S, von Wendt G, Hamsten A, et al (2009) Peripheral sensory neuropathy associates with micro- or macroangiopathy: results from a population-based study of type 2 diabetic patients in Sweden. Diabetes Care 32: 317–322. 10.2337/dc08-1250 19033412 PMC2628701

[8] Thrainsdottir S, Malik RA, Rosen I, Jakobsson F, Bakhtadze E, Petersson J, et al (2009) Sural nerve biopsy may predict future nerve dysfunction. Acta Neurol Scand 120: 38–46. 10.1111/j.1600-0404.2008.01118.x 19154542

[9] Dahlin LB, Granberg V, Rolandsson O, Rosen I, Dahlin E, Sundkvist G. (2011) Disturbed vibrotactile sense in finger pulps in patients with Type 1 diabetes—correlations with glycaemic level, clinical examination and electrophysiology. Diabet Med 28: 1045–1052. 10.1111/j.1464-5491.2011.03328.x 21843302

[10] Nelander J, Speidel T, Bjorkman A, Dahlin LB (2012) Vibration thresholds are increased at low frequencies in the sole of the foot in diabetes-a novel multi-frequency approach. Diabet Med 29: e449–456. 10.1111/dme.12024 22998552

[11] Stromberg T, Dahlin LB, Lundborg G (1998) Vibrotactile sense in the hand-arm vibration syndrome. Scand J Work Environ Health 24: 495–502. 9988092 10.5271/sjweh.374

[12] Louraki M, Karayianni C, Kanaka-Gantenbein C, Katsalouli M, Karavanaki K (2012) Peripheral neuropathy in children with type 1 diabetes. Diabetes Metab 38: 281–289. 10.1016/j.diabet.2012.02.006 22503144

[13] Blankenburg M, Kraemer N, Hirschfeld G, Krumova EK, Maier C, Hechler T, et al (2012) Childhood diabetic neuropathy: functional impairment and non-invasive screening assessment. Diabet Med 29: 1425–1432. 10.1111/j.1464-5491.2012.03685.x 22507184

[14] Olsen BS, Nir M, Kjaer I, Volund A, Mortensen HB (1994) Elevated vibration perception threshold in young patients with type 1 diabetes in comparison to non-diabetic children and adolescents. Diabet Med 11: 888–892. 7705028 10.1111/j.1464-5491.1994.tb00374.x

[15] O'Brien JC, Williams HG, Bundy A, Lyons J, Mittal A (2008) Mechanisms that underlie coordination in children with developmental coordination disorder. J Mot Behav 40: 43–61. 10.3200/JMBR.40.1.43-61 18316296

[16] Lundstrom R, Stromberg T, Lundborg G (1992) Vibrotactile perception threshold measurements for diagnosis of sensory neuropathy. Description of a reference population. Int Arch Occup Environ Health 64: 201–207. 1328063 10.1007/BF00380910

[17] Gescheider GA, Bolanowski SJ, Hall KL, Hoffman KE, Verrillo RT (1994) The effects of aging on information-processing channels in the sense of touch: I. Absolute sensitivity. Somatosens Mot Res 11: 345–357. 7778411 10.3109/08990229409028878

[18] Verrillo RT (1980) Age related changes in the sensitivity to vibration. J Gerontol 35: 185–193. 7410775 10.1093/geronj/35.2.185

[19] Hilz MJ, Axelrod FB, Hermann K, Haertl U, Duetsch M, Neuendörfer B. (1998) Normative values of vibratory perception in 530 children, juveniles and adults aged 3–79 years. J Neurol Sci 159: 219–225. 9741411 10.1016/s0022-510x(98)00177-4

[20] Meh D, Denislic M (1995) Influence of age, temperature, sex, height and diazepam on vibration perception. J Neurol Sci 134: 136–142. 8747856 10.1016/0022-510x(95)00230-9

[21] http://www.iso.org/iso/catalogue_detail.htm?csnumber=27716.

[22] Schlee G, Sterzing T, Milani TL (2009) Foot sole skin temperature affects plantar foot sensitivity. Clin Neurophysiol 120: 1548–1551. 10.1016/j.clinph.2009.06.010 19616994

[23] Verrillo RT, Bolanowski SJ (2003) Effects of temperature on the subjective magnitude of vibration. Somatosens Mot Res 20: 133–137. 12850822 10.1080/089902203100105163

[24] Lundborg GN, Bjorkman AC, Rosen BN, Nilsson JA, Dahlin LB (2010) Cutaneous anaesthesia of the lower leg can improve sensibility in the diabetic foot. A double-blind, randomized clinical trial. Diabet Med 27: 823–829. 10.1111/j.1464-5491.2010.03014.x 20636964

[25] Lundborg G, Dahlin LB, Lundstrom R, Necking LE, Stromberg T (1992) Vibrotactile function of the hand in compression and vibration-induced neuropathy. Sensibility index—a new measure. Scand J Plast Reconstr Surg Hand Surg 26: 275–279. 1335164 10.3109/02844319209015271

[26] Wikland KA, Luo ZC, Niklasson A, Karlberg J (2002) Swedish population-based longitudinal reference values from birth to 18 years of age for height, weight and head circumference. Acta Paediatr 91: 739–754. 12200898 10.1080/08035250213216

[27] Chemnitz A, Bjorkman A, Dahlin LB, Rosen B (2013) Functional outcome thirty years after median and ulnar nerve repair in childhood and adolescence. J Bone Joint Surg Am 95: 329–337. 10.2106/JBJS.L.00074 23426767

[28] Inami K, Chiba K, Toyama Y (2005) Determination of reference intervals for vibratory perception thresholds of the lower extremities in normal subjects. J Orthop Sci 10: 291–297. 15928892 10.1007/s00776-005-0897-5

[29] Meier PM, Berde CB, DiCanzio J, Zurakowski D, Sethna NF (2001) Quantitative assessment of cutaneous thermal and vibration sensation and thermal pain detection thresholds in healthy children and adolescents. Muscle Nerve 24: 1339–1345. 11562914 10.1002/mus.1153

[30] Lundstrom RJ (1986) Responses of mechanoreceptive afferent units in the glabrous skin of the human hand to vibration. Scand J Work Environ Health 12: 413–416. 3775331

[31] Gardner EP, Martin JH, Jessell TM (2000) Coding of sensory information In: Kandel ER, Schwartz JH, Jessell TM, editors. Principles of neural science. New York: McGraw-Hill pp. 411–429.

[32] Aaberg ML, Burch DM, Hud ZR, Zacharias MP (2008) Gender differences in the onset of diabetic neuropathy. J Diabetes Complications 22: 83–87. 10.1016/j.jdiacomp.2007.06.009 18280437

[33] Booya F, Bandarian F, Larijani B, Pajouhi M, Nooraei M, Lotfi J. (2005) Potential risk factors for diabetic neuropathy: a case control study. BMC Neurol 5: 24 16336693 10.1186/1471-2377-5-24 PMC1343576

[34] Thomsen NO, Englund E, Thrainsdottir S, Rosen I, Dahlin LB (2009) Intraepidermal nerve fibre density at wrist level in diabetic and non-diabetic patients. Diabet Med 26: 1120–1126. 10.1111/j.1464-5491.2009.02823.x 19929990

[35] Schlee G, Neubert T, Worenz A, Milani TL (2012) Children with ADHD show no deficits in plantar foot sensitivity and static balance compared to healthy controls. Res Dev Disabil 33: 1957–1963. 10.1016/j.ridd.2012.05.020 22728606

[36] Williams CM, Tinley P, Curtin M, Nielsen S (2012) Vibration perception thresholds in children with idiopathic toe walking gait. J Child Neurol 27: 1017–1021. 10.1177/0883073811432748 22433426

[37] Louraki M, Tsentidis C, Kallinikou D, Katsalouli M, Kanaka-Gantenbein C, Kafassi N, et al (2013) Reproducibility of vibration perception threshold values in children and adolescents with type 1 diabetes mellitus and associated factors. Prim Care Diabetes. 10.1016/j.pcd.2013.11.002 24315733

